# Artificial Intelligence Transforming Post-Translational Modification Research

**DOI:** 10.3390/bioengineering12010026

**Published:** 2024-12-31

**Authors:** Doo Nam Kim, Tianzhixi Yin, Tong Zhang, Alexandria K. Im, John R. Cort, Jordan C. Rozum, David Pollock, Wei-Jun Qian, Song Feng

**Affiliations:** 1 Biological Sciences Division, Pacific Northwest National Laboratory, 902 Battelle Blvd, Richland, WA 99352, USAjordan.rozum@pnnl.gov (J.C.R.); david.pollock@cuanschutz.edu (D.P.); weijun.qian@pnnl.gov (W.-J.Q.); 2 National Security Directorate, Pacific Northwest National Laboratory, 902 Battelle Blvd, Richland, WA 99352, USA; 3 Department of Biochemistry and Molecular Genetics, University of Colorado School of Medicine, Aurora, CO 80045, USA

**Keywords:** artificial intelligence, deep learning, machine learning, Post-Translational Modification

## Abstract

Post-Translational Modifications (PTMs) are covalent changes to amino acids that occur after protein synthesis, including covalent modifications on side chains and peptide backbones. Many PTMs profoundly impact cellular and molecular functions and structures, and their significance extends to evolutionary studies as well. In light of these implications, we have explored how artificial intelligence (AI) can be utilized in researching PTMs. Initially, rationales for adopting AI and its advantages in understanding the functions of PTMs are discussed. Then, various deep learning architectures and programs, including recent applications of language models, for predicting PTM sites on proteins and the regulatory functions of these PTMs are compared. Finally, our high-throughput PTM-data-generation pipeline, which formats data suitably for AI training and predictions is described. We hope this review illuminates areas where future AI models on PTMs can be improved, thereby contributing to the field of PTM bioengineering.

## 1. Introduction of Post-Translational Modification

### 1.1. Definition of Post-Translational Modification

**Figure 1:**
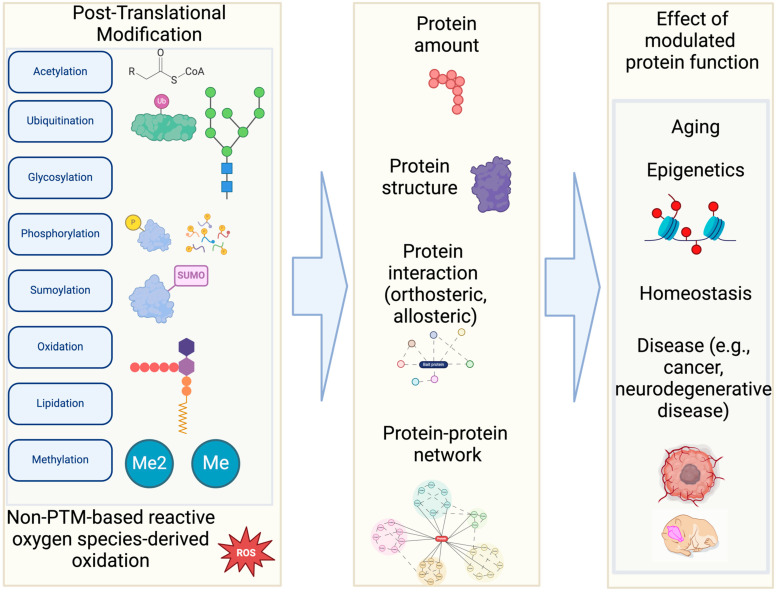
Various types of Post-Translational Modifications and their effects. This illustration shows several common types of PTMs out of 400 different types. Although these PTMs are often less represented in many computational modeling programs, they control various cellular activities. This figure is generated by BioRender and NIH BIOART [12].

Post-Translational Modifications (PTMs) are covalent alterations to one or more amino acids (AAs) that occur after translation from mRNA to polypeptide chains [1]. These alterations often occur on polar AAs, as well as non-polar residues at the N-termini of proteins [2]. There are more than 400 known types of PTMs, but the most abundant are phosphorylation, glycosylation, acetylation, methylation, and ubiquitin/ubiquitin-like modifications (Figure 1) [1,3,4,5]. In addition, oxidation of methionine and thiol-oxidation of cysteine [6] have been studied as prevalent PTMs in many different organisms.

PTMs come in two forms: reversible/irreversible covalent side chain edits and irreversible peptide backbone cleavage (i.e., proteolytic cleavage) [7]. Changes to side chains (e.g., glycosylation, phosphorylation, and methylation) are either reversible or irreversible, and many residues on a single protein may have side chain modifications, and these side chains can be modified more than once [5].

PTMs may affect the shape and electrostatic properties of the modified residues, therefore having important implications on protein structures and functions, such as expression [8], degradation, protein–protein interaction, catalytic activity, conformational change, and binding to DNA or RNA (Figure 1) [4]. For example, phosphorylation refers to the adding of a phosphoryl group to the side chain of an AA. The phosphoryl group is usually transferred from a donor such as a nucleotide triphosphate or other phosphoryl donor by protein kinases and may be removed by phosphatases [9]. Phosphorylation commonly occurs on serine, threonine, and tyrosine residues. Acetylation can also occur non-specifically, in response to epigenetics regulation, protein damage, aging processes [10]—particularly when there is an excess of acetyl-CoA or acetyl phosphate present. In epigenetic regulation, lysine acetylation on histones influences DNA accessibility and chromatin compaction. Cysteine may undergo PTMs such as nitrosylation, sulfenylation, and glutathionylation. Therefore, along with other physiological factors (e.g., body temperature, infection, inflammation), PTMs contribute to explaining how a limited number of genes can result in a wide range of proteoforms [11] that expand phenotypic diversity [5].

### 1.2. Importance of Post-Translational Modification

A substantial portion of PTMs can occur incidentally and do not lead to significant functional changes. This is understandable, as most PTMs minimally alter protein structures. For instance, the majority of backbone conformation changes due to phosphorylation are small (median root mean squared deviation-RMSD of 1.1 Å). Only 13% of phosphorylation events cause a structural change greater than 2 Å RMSD; even when focusing on backbone changes, only 28% result in a structural shift exceeding 2 Å RMSD [13].

When PTMs affect protein function, their impact is significant [14], and PTMs that cause larger structural changes tend to correlate more strongly with functional changes [13]. For instance, PTMs are essential for regulating every stage of the nicotinic acetylcholine receptor life cycle, encompassing receptor expression, membrane stability, and function [15]. As a result, PTMs are frequently targeted for disease treatment and detection. For example, cancer therapies have been developed to control the addition or removal of PTMs contributing to the disease [2]. From a metabolic perspective, understanding the role of mitochondria-related PTMs in tumorigenesis is anticipated to guide new approaches for next-generation cancer therapies, as PTMs play a pivotal role in regulating mitochondrial functions in cancer [16].

PTMs can also serve as biomarkers for disease detection. For example, elevated hemoglobin A1C levels are commonly used to diagnose and monitor diabetes management. Hemoglobin A1C is glycosylated when exposed to glucose in the blood, and high blood glucose levels in diabetes result in increased hemoglobin A1C levels [3]. Another example is the use of PTM-related enzymes as biomarkers in glioblastoma multiforme [17].

Given their many uses in clinical and molecular contexts, there has been much effort put towards PTM site discovery and characterizing their respective functions. In particular, irregular phosphorylation is one of the mechanisms underlying the development of many cancers [9,18]. Thus, PTMs are an important subject of study in understanding cellular and molecular regulatory systems.

### 1.3. Significance of Artificial Intelligence on Post-Translational Modification Research

Computational modeling for PTMs involves multiple complex factors. For instance, whether a sequon is N-glycosylated depends on various factors such as the distance to the next glycosylation site and the surrounding sequences. The need to incorporate complex factors into structural modeling for PTM prediction arises because most PTMs—including phosphorylation—induce only small-scale, stabilizing conformational changes by modulating local residue fluctuations through conformational selection [13]. However, there is a vast number of residues with PTMs, and they are often unstable and low in abundance. Therefore, it has been challenging to conduct large-scale identification and functional characterization using conventional lab methods such as mass spectrometry [19]. Consequently, deep learning (DL) approaches, including protein space embedding large language models, can address these complexities more effectively than traditional machine learning (ML) or shallow neural network models [20]. Thus, DL approaches with large datasets are particularly valuable for extracting meaningful insights related to PTM [20,21]. These methods allow us to use combinations of sequence, structural, and other information to identify residues that may harbor PTMs and their possible implications on function.

Therefore, in this review, we discuss recent advancements in structural and sequence-based PTM research leveraging artificial intelligence (AI), explore related non-canonical amino acid studies, and examine experimental data generation as well as the utilization of current PTM databases (DBs).

## 2. Computational Modeling for PTM Research

### 2.1. Structural Modeling for PTM Prediction

PTMs often occur on polar residues and non-polar residues near the N-termini. Thus, protein sequence is the most basic input in many computational tools to predict PTM location and function. In addition, some sequons with conserved motifs also give information on where PTMs may be located. For example, the N-X-[S/T] sequon is the site in which glycosylation occurs [20].

However, sequence information is not always sufficient to predict PTM site location or function. Structural information is also crucial for PTM modeling. For example, tools like StructureMap [22] and a specific phosphorylation site prediction program (i.e., PhosAF) [10] suggest that a variety of structural factors (e.g., solvent accessible surface area, dynamic region of structure represented by a predicted local distance difference test—pLDDT) are crucial for understanding the preferred locations and functions of PTMs. These structural requirements are logical, as exposed areas are typically more functionally relevant [13,23] and accessible to “writers” that introduce PTMs. Additionally, pLDDT scores can help identify short intrinsically disordered regions (IDRs), where PTMs are more frequently located. Moreover, conserved sequences alone do not ensure N-linked glycosylation, as many motifs or sequons are buried, making them inaccessible to glycosylation enzymes [20].

In addition to rudimentary sequence and structural information, other advanced annotations have been integrated into PTM models. For example, the FuncPhos-STR tool integrates phosphosite sequence evolution and protein–protein interaction information into structural information from AlphaFold2 [24]. In addition, structural deep network embedding [25] was employed to transform the high-dimensional structural data of a protein–protein interaction network into a more manageable low-dimensional space.

#### 2.1.1. PTM Structural Map

StructureMap analyzed structural trends of extensive lists of PTMs (i.e., phosphorylation, ubiquitination, sumoylation, acetylation, methylation, and glycosylation) [22]. Their analyses have shown that a significant number of phosphorylation sites are located within IDRs, many of which comprise the activation loops of kinases. This suggests a functional relevance to the flexible nature of these regions in protein phosphorylation. However, some analyses of these findings may have been at least slightly biased depending on the choice of datasets used. For example, certain datasets are derived from samples treated under specific conditions. Moreover, PTM sites in physically less accessible regions may have been underrepresented.

#### 2.1.2. Structural Simulation to Study Non-Canonical Amino Acid Effects

PTMs can be considered naturally occurring types of non-canonical amino acids (ncAAs). With much larger chemical space than canonical amino acids (cAA) [26], ncAAs allow more possibilities of protein and peptide engineering [27]. For example, incorporations of ncAAs increased protease resistance [28], membrane permeability [29], and peptide binding affinity [30]. Therefore, various computational approaches have been developed to enhance the utility of ncAAs. For example, in silico screening with the Random Non-standard Peptide Integrated Discovery platform enhances library diversity and enables the discovery of diverse peptide scaffolds containing multiple ncAAs. This approach is particularly useful for identifying mutations possible at position 1 in thioether macrocycle discovery. It also removes the need to create multiple libraries with varying initiators, streamlining the process. Additionally, after the seminal paper of Rosetta-based ncAA rotamer library constructions [31], various computational designs [32,33,34] (e.g., an ncAA probe to study protein–peptide interactions [26] and peptide cylicization [35]) and force field developments [36] have been made. For example, Renfrew et al. have shown that replacing phenylalanine with 4-methyl-phenylalanine at the protein–peptide interface improved binding affinity by 2-fold [31]. Similarly, replacing non-fluorine AAs with fluorine AAs in the core region of helix bundle protein improved its thermal stability [37]. Designing peptides/proteins that are resistant to enzymatic degradation is important for successful biomarker effectiveness and more stable vaccine delivery. Therefore, there have been replacements of some L-AAs with D-AAs to improve peptide stability against protease [38,39,40]. Additionally, mirror images of all protein structures in Protein Data Bank [41] have been generated with the hope of providing potential drug leads [42]. However, D-AA substitutions in the middle of a peptide may disrupt surface topology, secondary structure, and function of the original peptide with L-AAs [40,42].

Force fields (FFs) for ncAAs have not been thoroughly developed for semi-empirical quantum mechanical calculation, and recent extended tight-binding quantum chemistry methods [43] have been tested for cAAs only [44]. However, FFs for empirical methods have been developed. For example, Khoury et al. developed ab initio-derived AMBER FF03 compatible charge parameters for 147 ncAAs including β- and N-methylated AAs [36]. Since more accurate protein FFs have been developed, further FF development for ncAAs is expected. One example of a more accurate protein FF is cross-term map (CMAP); a grid-based correction for the protein φ- and ψ-angular dependence of the energy was added to CHARMM22 FF [45]. The CMAP correction was validated by removing substantial deviations from experimental backbone root-mean-square fluctuations and N-H NMR order parameters [46].

Protein side chains can adopt various conformations influenced by the protein backbone angles (i.e., φ and ψ) and interactions with neighboring residues. These conformations, determined by one or more χ angles, are referred to as rotamers. A collection of such conformations is known as a rotamer library. Sampling expected or favorable rotamers is especially important for the protein core regions and surface regions if they are dominated by electrostatic interactions. In 2012, Rosetta software suite added rotamer libraries of 114 ncAAs [31]. However, more than 200 ncAAs can be incorporated into proteins in prokaryotic and eukaryotic systems [47]. Since the possible combination of these ncAAs can be exponentially large theoretically, scientists have been depositing ncAA rotamers (e.g., residue_types + patches) into Rosetta (1571 as of this writing).

In addition to the side chain, different backbones can also be modeled. Non-canonical protein backbones, such as oligooxopiperazines, oligopeptoids (peptoids are peptidomimetic oligomers that mimic the motifs of protein secondary structures), β-peptides, hydrogen bond surrogate helices, and oligosaccharides, have been utilized for structure prediction and the design of non-peptidic oligomer scaffolds [48]. For example, the Bonneau group assessed peptoid foldamer conformation as a conventional AA rotamer search [49]. Schneider et al. designed peptoid–peptide macrocycles to inhibit the β-catenin T-cell factor interaction in prostate cancer [50]. These rotamers have been manually parameterized with MakeRotlib [31], which is preceded by quantum mechanical (QM) calculation, OpenBabel [51], and molfile_to_params_polymer.py [52]. However, more automated ncAA rotamer samplings were developed. For example, AutoRotLib generates parameters for an ncAA rotamer library from Simplified Molecular Input Line Entry System code [26]. Unfortunately, license fees associated with using this software can be a barrier for potential users. On the other hand, BioChemical Library [53] automatically generates ncAA rotamer libraries as an open-source program. This highlights a broader impact of open science, as emphasized in other DL review [54]. Developing accurate rotamer libraries is particularly crucial for longer amino acids, such as asparagine, which exhibit a larger number of rotamer conformations [31].

For PTM-specific ncAA simulations, molecular dynamics (MD) simulations have been used. For example, Li et al. used MD simulation and molecular mechanics generalized Born/surface area (MM-GBSA) binding free energy calculations to understand the influence of phosphorylation on death-associated protein kinase 1 activity [55]. Similarly, Mejia-Rodriguez et al. developed a PTM-specific force field with QM calculation and studied the impact of S-nitrosylation of several cysteines using MD and docking simulations [56]. Other docking simulations to model covalent bonding include CovPepDock, which incorporates covalent binding between the peptide and a receptor cysteine [57], covalent labeling-guided protein–protein docking in Rosetta [58], and Meeko-derived [59,60] AutoDock-GPU [61].

### 2.2. Deep Learning Approaches for PTM

**Figure 2:**
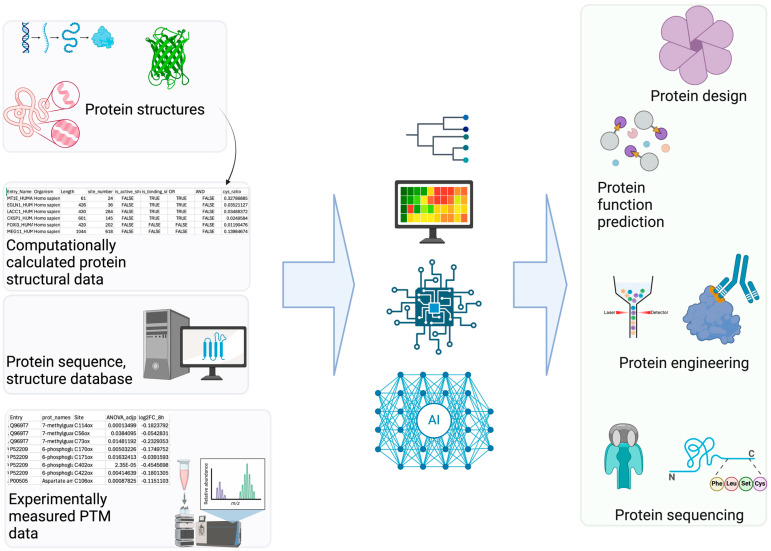
AI Applications in PTM Research. (**Left**): Various PTM-related datasets serve as the foundation for building AI/ML models. (**Right**): These models enable diverse applications utilizing accurate PTM information, such as predicting PTM sites and their associated functions [74].

Computational PTM modeling (including site, structure, and function prediction) is a complex problem with numerous contributing features. Additionally, there are many types of data (i.e., sequence, structure, metadata such as species) for PTM modeling. DL is well suited for PTM applications (Figure 2) with its advantage of leveraging large quantities of data effectively [62,63]. DL often performs self-supervised learning more effectively than non-DL ML methods. This advantage of DL stems from its ability to process complex non-linear properties through deeper layers in neural networks compared to other non-DL-based ML methods. Therefore, DL methods often outperform non-DL-based ML methods for PTM modeling [20]. Good examples of DL-based PTM structural modeling includes RosettaFold All-Atom [64] and AlphaFold3 [65]. These programs can predict covalently modified protein structures including PTMs using a denoising diffusion probabilistic model [61]. Therefore, these overcome the limitations of other protein structure prediction models (e.g., OmegaFold [66], Chai-1 [67], ESMFold [68]). Additionally, Meiler and colleagues have incorporated ML into PTM prediction [69]. Specifically, they trained a site prediction model using TensorFlow for 18 of the most abundant PTMs (e.g., glycosylation and phosphorylation) and made it interoperable with existing Rosetta protocols [70]. Other efforts in PTM structural modeling include fine-tuning AlphaFold Multimer to predict phosphopeptide–protein interactions [71].

FuncPhos-STR makes predictions about phosphosite function. This tool uses phosphosite sequence evolution data with protein–protein interaction information, which allows them to integrate information about the structure and dynamics of the protein into their DL model [24]. Other tools investigate how mutations affect PTM sites in protein structure. For example, MIND-S does this using a graph neural network with multi-head attention [72]. Cao et al. applied DL methods to advance nanopore-based protein sequencing, extending its capabilities to the PTM level [73]. In their approach, a peptide is passed through a nanopore, causing changes in the electrical current that correspond to the different AA residues traversing it. They utilized a long short-term memory (LSTM) recurrent neural network to interpret these electrical signals and a multi-layer perceptron (MLP) to predict the peptide sequences. This methodology allows them to associate specific current patterns with PTM predictions, as modified residues exhibit distinct electrical signatures compared to their non-modified counterparts.

#### 2.2.1. Language Models for PTM

Other recently developed PTM DL tools include the use of pre-trained protein language models (pLMs). PLMs use ML techniques typically developed to analyze human language to make context-specific predictions about protein structure and function [75,76]. Therefore, diverse protein-related language models have been developed [68,77,78,79]. For PTM research, LMPhosSite makes predictions on phosphosite locations. This tool integrates contextualized embeddings from a pLM to improve performance [80]. LMNglyPred also utilizes pLMs by repurposing embeddings from a pre-trained pLM to predict N-linked glycosylation sites. For this, the authors used an MLP for feature extraction with pre-trained per residue pLMs [20]. PTM-Mamba includes information from its previous pLMs in their model but also incorporates PTM tokens into training a pLM, improving its model’s accuracy for PTM-specific tasks [81]. Supervised word embeddings from a pLM (i.e., ProtT5) were also used to predict protein succinylation sites [82]. Recently, prompt-based fine-tuning of a GPT-2 model (i.e., PTMGPT2) was reported as an interpretable PTM prediction [83]. It identifies sequence motifs crucial for molecular recognition and analyzes the effects of mutations occurring at or near PTM sites, providing better insights into protein functionality.

#### 2.2.2. Comparison of Deep Learning Approaches for PTM

Meng et al. presented a comprehensive compilation of studies employing DL techniques for PTM in early 2022 [84]. Apart from the datasets they introduced, primary distinctions among these studies were evident: the methods employed for handling PTM data and the subsequent encoding or embedding strategies used to feed this data into the DL models, as well as the diverse DL model architectures employed. While some studies had already incorporated attention mechanisms, the adoption of transformer models for PTM tasks was still not widespread.

The types of input data are closely related to the encoding strategies employed in PTM research (mostly PTM site prediction) using DL models. Most studies utilize protein sequences as input, as seen in tools like MusiteDeep (Table 1) [85]. Some studies incorporate protein structural information, e.g., models such as MIND-S [72]. Others also include annotation information, like PTM-Mamba [81].

Encoding is the process of converting biological information into a numerical format that can be processed by DL models. For protein sequences, major encoding methods include one-hot encoding (or one-of-k encoding), where each AA is represented as a binary vector with a length equal to the number of possible AAs. Only one position in the vector is set to 1, corresponding to the specific AA, while all other positions are set to 0. Various embedding methods are also widely used, including embedding layers integrated within DL models that transform AA sequences into continuous vector spaces, capturing contextual relationships. Word embedding techniques, such as Word2Vec or FastText, treat AAs or k-mers as words and generate embeddings based on their contextual similarity. Pre-trained protein models like ProtT5 provide embeddings that capture rich contextual information about protein sequences, learned from large-scale protein datasets [76]. Although BLOSUM62, a substitution matrix that scores alignments between protein sequences, has been used historically, its popularity has declined. When the data includes evolutionary information, Position-Specific Scoring Matrices (PSSMs) can be employed [86]. PSSMs are generated from multiple sequence alignments and reflect the evolutionary conservation of AA residues at each position. This encoding method has been utilized by PTM models (i.e., DeepAcet) and a general protein structure prediction and design program (i.e., transform-restrained Rosetta, which is abbreviated as trRosetta) [87,88].

MLPs have historically been the initial approach in the field of PTM prediction, serving as a reliable baseline method, e.g., DL-based protein lysine acetylation modification prediction (Deep-PLA) and Histone-Net [89,90]. However, more complex neural network architectures, such as CNNs and RNNs, specifically LSTMs, have gained prominence, such as models like DeepPhos, MusiteDeep, and LMPhosSite [80,85,91]. CNNs excel at detecting local patterns within sequences, which is particularly useful for identifying conserved motifs or sequence motifs around PTM sites. Their ability to learn features through convolutional layers allows them to detect motifs regardless of their position in the input sequence, making CNNs robust to slight shifts in sequence position. The layered architecture of CNNs enables them to build hierarchical representations of the input data, where lower layers capture simple patterns such as small motifs, and higher layers capture more complex patterns like secondary structure elements. LSTMs, on the other hand, are designed to handle sequential data and can capture long-range dependencies within protein sequences. This capability is crucial for PTM prediction, where the modification site might depend on residues that are far apart in the sequence. Additionally, bidirectional LSTMs can consider information from both former and latter residues, providing a more comprehensive understanding of the sequence context. When protein structural information is available, Graph Neural Networks (GNNs) also represent a highly effective choice, such as MIND-S [72]. GNNs can model the complex relationships between residues in a protein structure, capturing the three-dimensional spatial arrangements and interactions that are crucial for understanding protein function and PTMs.

Recently, transformers have gained popularity in this field, such as TransPTM [92]. The advantages of transformers in this context include their ability to capture longer-range dependencies than LSTM, their parallel processing capabilities, and their scalability with large datasets. Transformers excel at capturing long-range dependencies within protein sequences using the self-attention mechanism. This mechanism allows each position in the sequence to directly attend to all other positions, effectively capturing relationships between distant residues. Hence, it allows the modeling of even proteins with high contact order. The parallel processing capability of transformers is another significant advantage. Traditional LSTMs can be computationally intensive and slow, especially for long sequences. In contrast, transformers process all positions in the sequence simultaneously, leading to more efficient training and inference. Pre-training transformer models (like ProtT5) on large protein datasets allows them to learn rich, generalizable representations of protein sequences. These pre-trained models can then be fine-tuned on specific PTM prediction tasks.

## 3. Experimental Data for PTM ML

### 3.1. Mass Spectrometry-Based PTM Proteomics for ML

Mass spectrometry-based proteomics is the preferred technology for providing large-scale and unbiased PTM measurements suitable for ML. Due to the unique chemistries and usually low-abundance nature of many PTMs, specific sample processing workflows and MS data acquisition methods have been developed for each PTM of interest. In general, these approaches aim to provide high coverage of the PTMome, precise localization of the modification site, and accurate quantification of the level of modification. To reach high PTMome coverage, selective enrichment and fractionation are often used prior to MS analysis. For example, peptides were fractionated by basic reversed-phase liquid chromatography (bRPLC), and each fraction was enriched by immobilized metal affinity chromatography for phosphoproteomics [93]. In the case of redox modifications, our group developed resin-assisted capture to enrich oxidized cysteines first, followed by bRPLC to achieve in-depth profiling of the redox proteome [94]. These approaches usually employ data-dependent acquisition to collect MS data and require a long instrument time due to extensive fractionation. Recently, data-independent acquisition (DIA) methods have been evaluated for PTM works, with some promising results showing the identification of >30,000 phosphorylation sites using a short single-shot LC–MS run [95]. In addition, precise localization of PTM sites is important because PTM events are site-specific, and knowledge of the modification site is critical for subsequent PTM-based engineering. MS data contains sequence-level information (e.g., b and y fragment ions in MS/MS spectra) and thus can be used to infer the site localization of modified AAs. Many algorithms have been developed for PTM site localization [96,97], and an interesting recent trend is to use DL-based framework to control the false localization rate [98]. Lastly, high-quality quantitative PTMomics data is required to enable ML to identify PTM signatures contributing a particular biological phenotype. Both isobaric tagging-based methods and label-free approaches are widely used in quantitative proteomics. Since a large sample size is beneficial for ML, isobaric tagging-based methods such as tandem mass tags are advantageous because of the sample multiplexing power. However, a shorter LC–MS run time coupled with advanced DIA approaches also provides an alternative for high-throughput analysis of hundreds of PTMomes for future ML studies.

### 3.2. Public PTM Databases

**Figure 3:**
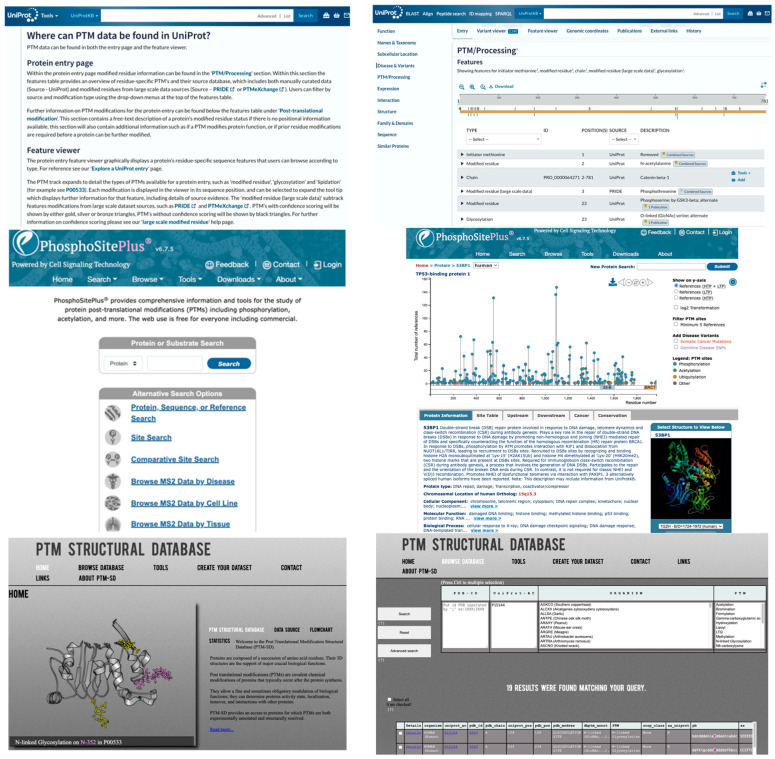
Representative PTM Databases. **Upper**: UniProt. **Middle**: PhosphoSitePlus. **Lower**: PTM-Structural Database. **Left**: input pages. **Right**: data retrieval pages.

To accelerate the application of DL in PTM research, leveraging publicly available PTM DBs, as previously summarized (Table 1 in Meng et al. [84]), is essential. Additional PTM DBs include GlycoEP datasets, which were compiled using sequence, evolutionary, and structural information [20]. Moreover, advancements in high-throughput proteomics have led to the development of extensive quantitative PTM proteome datasets, such as qPTM [99]. These datasets can also be integrated with other chemoproteomics DBs (e.g., CysDB) [100], further enhancing PTM research capabilities. Figure 3 shows three popular DBs (i.e., PhosphoSitePlus [101], UniProt [102], and PTM-Structural Database [103]). They offer various search options, including protein or substrate name, specific sites, disease, cell line, and tissue, and provide details such as the number of references for each PTM, along with the corresponding residue number, PDB ID, and UniProt ID.

## 4. Discussion

It is expected that AI-accelerated PTM study will propagate to other fields including more clinically relevant therapeutics and evolution research [104,105]. However, most current applications of ML and DL in PTM research have predominantly centered on predicting PTM sites [106]. This trend has been found for both mass spectrometry-derived proteomics data [96,97,98] and general protein structure and sequence data [20,24]. Predicting PTM sites holds significant values, as specific PTMs can bring about orthosteric or allosteric regulation in signal transduction [13,107]. For example, phosphorylation often exerts allosteric effects that extend well beyond the immediate vicinity of the phosphorylation site [13]. However, other aspects of ML/DL-assisted PTM research—such as nanopore-based PTM detection [73] and direct functional prediction [24,108]—remain relatively underexplored and require further investigation. Predicting PTM-induced functions will naturally involve structural studies, as larger conformational changes are generally more functionally relevant, particularly for many phosphorylation events [13].

Additionally, more structural biology data from methods like NMR [109,110] would be beneficial in these new research directions. PTM data is currently underrepresented in structural biology databases [13]. Moreover, advanced structural modeling that incorporates a conformational selection approach—often elucidated by techniques like NMR or cryo-EM single particle analysis—will provide a more accurate explanation for PTMs such as phosphorylation, which aligns more with conformational selection than with induced-fit structural rearrangement [13]. Expanding these efforts will enhance the application of PTM research, especially considering that, for phosphorylation alone, over 100,000 PTM sites have been identified, yet the enzymes regulating these sites are known for only a small fraction, and even fewer functions have been elucidated [111].

## 5. Conclusions

PTM studies are contributing significantly to the development of therapeutic and diagnostic tools for diseases such as cancer, metabolic disorders, and neurodegenerative conditions. As the field progresses, AI-driven PTM research is expected to drive innovations in clinical applications and evolutionary biology, transforming understanding of protein regulation and cellular systems. Therefore, we have discussed the significant role of AI in advancing PTM research, and the benefits of generating high-throughput PTM data for AI training. Additionally, advanced machine learning architectures, such as language models and graph neural networks, will continue uncovering novel aspects of PTM biology, while public DBs and collaborative efforts continue to expand the accessibility and scope of this endeavor. We expect that the combination of AI-driven predictions and more experimental structural biology data will further elucidate the molecular mechanisms underlying PTM-induced conformational changes and functional impacts.

## Figures and Tables

**Table 1:** Publicly accessible PTM modeling programs.

Year	Program Name	PTM Type	Model	Website
2024	LMNglyPred	Glycosylation	pLM	https://github.com/KCLabMTU/LMNglyPred (accessed on 28 December 2024)
2024	PTM-Mamba	Multiple	pLM	https://github.com/programmablebio/ptm-mamba (accessed on 28 December 2024)
2024	Sitetack	Multiple	CNN	https://sitetack.net (accessed on 28 December 2024)
2024	TransPTM	Acetylation	Transformer	https://github.com/TransPTM/TransPTM (accessed on 28 December 2024)
2023	MIND-S	Multiple	GNN	https://zenodo.org/records/7659116 (accessed on 28 December 2024)
2022	LMPhosSite	Phosphorylation	pLM, CNN	https://github.com/KCLabMTU/LMPhosSite (accessed on 28 December 2024)
2021	ScanSite 4.0	Phosphorylation	-	https://scansite4.mit.edu (accessed on 28 December 2024)
2020	MusiteDeep	Multiple	CNN	https://www.musite.net (accessed on 28 December 2024)
2019	DeepAcet	Acetylation	MLP	https://github.com/Lab-Xu/DeepAcet (accessed on 28 December 2024)
2019	DeepHistone	Multiple	CNN	https://github.com/QijinYin/DeepHistone (accessed on 28 December 2024)
2019	DeepPhos	Phosphorylation	CNN	https://github.com/USTC-HIlab/DeepPhos (accessed on 28 December 2024)
2019	Deep-PLA	Acetylation	MLP	http://deeppla.cancerbio.info (accessed on 28 December 2024)
